# High-throughput physical phenotyping of cell differentiation

**DOI:** 10.1038/micronano.2017.13

**Published:** 2017-05-08

**Authors:** Jonathan Lin, Donghyuk Kim, Henry T. Tse, Peter Tseng, Lillian Peng, Manjima Dhar, Saravanan Karumbayaram, Dino Di Carlo

**Affiliations:** 1 Department of Bioengineering, University of California, Los Angeles, CA 90095, USA; 2 CytoVale Inc., 384 Oyster Point Boulevard #7 South, San Francisco, CA 94080, USA; 3 Department of Biomedical Engineering, Tufts University, Medford, MA 02155, USA; 4 Eli and Edythe Broad Center of Regenerative Medicine and Stem Cell Research, Los Angeles, CA 90095, USA; 5 California NanoSystems Institute, Los Angeles, CA 90095, USA; 6 Jonsson Comprehensive Cancer Center, Los Angeles, CA 90095, USA; 7 Department of Mechanical Engineering, University of California, Los Angeles, CA 90095, USA

**Keywords:** cell mechanics, deformation, deformation kinetics, morphology, physical phenotype, stem cells

## Abstract

In this report, we present multiparameter deformability cytometry (m-DC), in which we explore a large set of parameters describing the physical phenotypes of pluripotent cells and their derivatives. m-DC utilizes microfluidic inertial focusing and hydrodynamic stretching of single cells in conjunction with high-speed video recording to realize high-throughput characterization of over 20 different cell motion and morphology-derived parameters. Parameters extracted from videos include size, deformability, deformation kinetics, and morphology. We train support vector machines that provide evidence that these additional physical measurements improve classification of induced pluripotent stem cells, mesenchymal stem cells, neural stem cells, and their derivatives compared to size and deformability alone. In addition, we utilize visual interactive stochastic neighbor embedding to visually map the high-dimensional physical phenotypic spaces occupied by these stem cells and their progeny and the pathways traversed during differentiation. This report demonstrates the potential of m-DC for improving understanding of physical differences that arise as cells differentiate and identifying cell subpopulations in a label-free manner. Ultimately, such approaches could broaden our understanding of subtle changes in cell phenotypes and their roles in human biology.

## INTRODUCTION

Cell physical properties, including the shape, size and resistance of cells to an applied load, stem from other structural and molecular cell properties in a complex manner that is not easily discerned^1–4^. Compared to the conventional molecular biomarkers, these physical properties integrate many molecular changes. Recent findings have clearly demonstrated that physical and mechanical properties can be a promising alternative for phenotyping a range of cell types in different stages. For example, a recent study identified that the differentiation potential of mesenchymal stromal/stem cells is strongly dependent on their elastic and viscoelastic properties^5^. Similarly, it was shown that cell mechanical markers can be a promising alternative for predicting osteogenesis of differentiating stem cells^6^. Other work has demonstrated that physical changes are important for explaining natural phenomena such as leukocyte demargination^7^.

Obtaining quantitative and reliable measurements of mechanical properties from a large population of cells has always been a challenge^8^. The conventional approaches have been atomic force microscopy or micropipette aspiration^9,10^, both of which provide a reliable measurement of the effective Young’s modulus of individual cells. However, both methods are time and labor intensive (tens of cells per hour), posing challenges for examining large populations of cells to either obtain statistically valid conclusions or identify rare sub-populations. Recent advances in micro-/nano-fabrication technologies have opened up a range of new mechanophenotyping technologies that can measure deformations of tens to hundreds of cells per second^11–19^. We recently reported a technology, called deformability cytometry, in which a cross-slot microfluidic channel is employed to generate a hydrodynamic extension zone where individual cells are exposed to uniform hydrodynamic stress and deformed^20^. Using a standard inverted microscope setup equipped with a high-speed camera, the technology successfully monitored cell size and deformation at a throughput of ~2000 cells per second. The developed technology, with cell size and deformability alone, successfully discriminated activated/non-activated leukocytes and identified malignancy in pleural effusion samples^21^; however, it was also noted that there were potentially other easily extracted parameters (e.g., time-dependent deformation and cell morphology) that might provide additional physical phenotypic information about cell type/status. As such, we hypothesized that expanding the analysis to additional physical properties may help to distinguish a spectrum of changes that occur as stem cells differentiate.

We were able to extract additional metrics from high-speed video that yielded repeatable cell type-specific values and distributions. Our results indicate that the additional parameters are important; particularly, the addition of morphological parameters (e.g., surface roughness and cell shape) significantly improved the classification accuracy when comparing induced pluripotent stem cells (iPSCs) vs. retinal pigmented epithelial cells (RPEs), neural stem cells (NSCs) vs. neurons and mesenchymal stem cells (MSCs) vs. osteocytes. In addition, iPSCs and RPEs were found to be the most physically distinguishable cell types, while MSCs and osteocytes were the least distinguishable. All of our results indicate that physical properties are modulated in stem cell differentiation and, thus, may play critical roles in cell physiology and can be used to identify cell populations.

## MATERIALS AND METHODS

### Deformability cytometry

**Figure 1:**
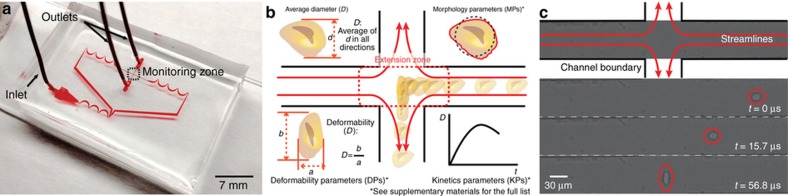
Deformability cytometry. (**a**) Microfluidic device with single inlet and two outlets. Asymmetric focusers and inertial focusing aid in biasing the cells to two vertically stacked equilibrium positions. (**b**) Cells are delivered uniformly to an extensional flow region where they are deformed. The deformation process is captured using high-speed photography, and parameters associated with size, morphology, strain or deformability, and strain rate are extracted from sequences of images through computer-automated image analysis. (**c**) Bright-field images of a cell entering the extensional flow and deforming.

The microfluidic device used for m-DC has been previously described (Figure 1)^20^. In brief, traditional soft lithography techniques were used to fabricate polydimethylsiloxane microfluidic chips in which cells are uniformly delivered to an extensional flow region (Figure 1). Cell suspensions (~100 000 cells per mL) were injected into the microfluidic chip using a syringe pump. Uniform delivery was achieved using inertial microfluidics with a channel aspect ratio of 2 in order to create two vertically stacked focusing positions (channel dimensions 60×30 μm, flow rate 750 μL min^−1^). The extensional flow caused cells to deform, and the process of deformation was captured using high-speed bright-field photography (~500 000 frames per second).

Automated computer analysis (MATLAB) was then performed to extract physical phenotype parameters from single cells. Prior to deformation, cell size and morphology were measured. Cell size metrics capture cell diameter and area, with cell area being robust to cell shape. Cell morphology metrics compare the distance from the cell membrane to the cell center (*r*_membrane_) with a moving average in order to describe the shape and surface roughness of a cell. For these measurements, a radial coordinate system was used to simplify calculations. During the deformation process, cell deformability and deformation kinetics were measured. Deformability captures the peak deformation of each cell normalized by size as an aspect ratio, and the deformation kinetics summarize the aspect ratio changes in the cell as a function of time. The resulting physical phenotype was composed of 21 parameters that fall under the broad categories of cell size, deformability, deformation kinetics, and morphology (Figures 1b, c, and Supplementary Table S1). General descriptions of the parameters are included in Equation (1).

(1){Size=∑θ=0360∘rmembrane(θ)Deformability,D=lverticallhorizontalMorphology=∑θ=0360∘[rmembrane(θ)−∑ϕ=θ−nθrmembrane(ϕ)]Kinetics=∑tD(t)−D(t−1)}

### Cell culture and preparation for measurement

iPSCs (1002) and RPEs were obtained through the stem cell core banks at the University of California, Los Angeles (Eli and Edythe Broad Center of Regenerative Medicine and Stem Cell Research), a funded research facility of the California Institute of Regenerative Medicine (CIRM). The iPSCs were derived from a punch biopsy from the normal human skin of a single patient, and details regarding the generation, culture, and characterization of the iPSCs have been previously published^22^. RPEs were derived from 1002 iPSCs at passage 6. 1002 iPSCs were plated in suspension in low-adherent dishes with 14% knockout serum replacement and 10 mM nicotinamide and cultured for 2 weeks. Then, activin A (final concentration of 140 ng mL^−1^) and fibroblast growth factor (final concentration of 20 ng mL^−1^) were added into the culture media, and cells were allowed to further grow for an additional 3–4 weeks. Later, pigmented regions of the embryoid bodies were manually dissected using a scalpel under a microscope and re-plated as adherent cultures in RPE media (Alpha Dulbecco’s Modified Eagle’s medium supplemented with 4% fetal bovine serum, 0.02 ng mL^−1^ triiodothyronine, 0.02 μg mL^−1^ hydrocortisone, 0.25 mg mL^−1^ taurine, 10 mM nicotinamide, non-essential amino acids, N1, 0.1 mM beta-mercaptoethanol, and Glutamax)^23^. Pigmented monolayers of cells were passaged both enzymatically and mechanically and plated at a density of 10 000 cells per cm^2^ (Supplementary Figure S1). Cultured cells were trypsinized for 3 min (0.025% trypsin, Sigma-Aldrich, St. Louis, MO, USA) and resuspended in phosphate-buffered saline prior to measurement.

Neural stem cells derived from iPSCs were acquired from XCell Science, Inc. (Novato, CA, USA). The NSCs were plated in microplates coated in extracellular matrix (Matrigel, Becton Dickinson, BD Biosciences, San Jose, CA, USA). They were cultured in neurobasal medium (Life Technologies, Carlsbad, CA, USA) supplemented with recombinant human fibroblast growth factor 2 (Stemgent, Lexington, MA, USA), B27 supplement (Life Technologies), GlutaMAX (Life Technologies), non-essential amino acids (Life Technologies) and 1% penicillin/streptomycin (Life Technologies). Cells were seeded at an initial density of 100 000 cells per cm^2^ and allowed to grow for 5 days with a passage on day 3 (Supplementary Figures S2 and S3). Cells were released from the surface for measurement using Accutase (Life Technologies) for 3–5 min until the cells were visibly detached. NSCs were then resuspended in growth medium for 30 min at room temperature prior to beginning the measurement.

Pre-differentiated neurons derived from iPSCs were acquired from XCell Science Inc. Neurons were plated on microplates coated with poly-L-ornithine (Sigma-Aldrich) and mouse laminin (Thermo Fisher Scientific, San Diego, CA, USA) at a density of 50 000 cells per cm^2^. They were cultured in proprietary medium supplied by XCell Science, Inc. for 5 days (Supplementary Figures S2 and S3). Cells were released from the microplate for measurement using Accutase (Life Technologies) for ~5 min until the cells were visibly detached. The neurons were then resuspended in growth medium for 30 min at room temperature prior to measurement.

Human adipose-derived stem cells (hMSCs, Thermo Fisher Scientific) were cultured in tissue culture flasks in MesenPRO RS medium (Thermo Fisher Scientific). Three separate vials of cells were purchased, each derived from a single donor and received at passage number 1. Cells were seeded at ~5000 cells per cm^2^ and were allowed to grow for 17 days. Cells were released for measurement using TrypLE Express (Thermo Fisher Scientific) for ~7 min. hMSCs were resuspended in growth medium for 30 min at room temperature prior to measurement. The effect of m-DC measurements on hMSC viability and differentiation was also evaluated (Supplementary Figures S4–S6). In addition, the batch-to-batch variation of hMSCs was assessed using DC (three vials of hMSCs were compared with data pooled from three technical replicates per sample, Supplementary Figure S7).

Human osteocytes were derived from hMSCs using the StemPro Osteogenesis Differentiation Kit (Thermo Fisher Scientific). hMSCs were initially seeded as previously described and allowed to grow for 7 days in MesenPRO RS medium. They were then placed in osteogenesis differentiation medium for 10 days. Cells were released for measurement using TrypLE Express for ~7 min. Osteocytes were then resuspended in growth medium for 30 min at room temperature before measurement.

### Evaluation of parameter importance with an iterative support vector machine approach

To evaluate the importance of each physical parameter, support vector machines (SVMs) were trained to classify iPSCs vs. RPEs, NSCs vs. neurons, and MSCs vs. osteocytes. Each of these comparisons represents a differentiated cell type and a pluripotent progenitor. In brief, an SVM is a supervised machine-learning algorithm that defines a boundary based on training data to classify data points into one of two categories. Here we use SVMs as a tool to determine whether physical parameters enable us to classify cells into separate categories (or classes).

Initially, the SVMs were provided with two parameters, cell diameter and maximum deformation. This established a baseline accuracy based on metrics previously measured by deformability cytometry. Next, a new set of SVMs were trained on the full physical phenotype data set. The improvement in classification accuracy represents the cellular information captured by the new physical phenotype parameters. In each case, SVMs were trained on labeled populations of each cell type and then tested against a 1:1 unlabeled mixture of the two cell types. The classification accuracy was therefore defined as the percentage of cells that were correctly identified during the SVM test.

To evaluate the relative importance of different parameters, SVMs were incrementally trained, adding one parameter at a time. The training began with average cell diameter and maximum deformation, metrics collected by the original deformability cytometry system. From there, the parameters were added to the SVM sequentially to maximize classification accuracy. At each step in this process, a new set of SVMs were trained, each with the parameters selected in previous iterations as well as one of the remaining parameters. The addition of a remaining parameter that led to the best performance for the new SVM was then included in the next iteration. In this way, parameters that were more important for classifying the cells were added sooner in the process. This process quantitatively revealed the order in which parameters provide new independent information for distinguishing between the two cell types.

In all cases, a grid search was performed on a random subset of the data (500 data points from each cell population) to determine optimal training parameters (radial basis function kernel). Classification accuracy was determined by performing a five-fold cross validation training on a random subset of the data (5000 data points from each cell population).

### Visualization of physical phenotypes

A two-dimensional projection of the physical phenotype is produced using visual interactive stochastic neighbor embedding (viSNE), an algorithm that reduces dimensionality while preserving spatial relationships between data points^24^. This analysis was performed on iPSCs, NSCs, and neurons, three cell types that represent points along the spectrum of differentiation. A two-dimensional projection allows for the visualization of relationships between cell types and the changes in physical phenotype that occur during differentiation. Further exploration of the differences in physical phenotype between the cell types was performed by relabeling the projections using a parameter from each of the four broad categories of physical phenotypic parameters. Relabeling the projections in this way helped to reveal changes in physical phenotype that occur during differentiation as well as the variability among cells of the same type.

## RESULTS

**Figure 2:**
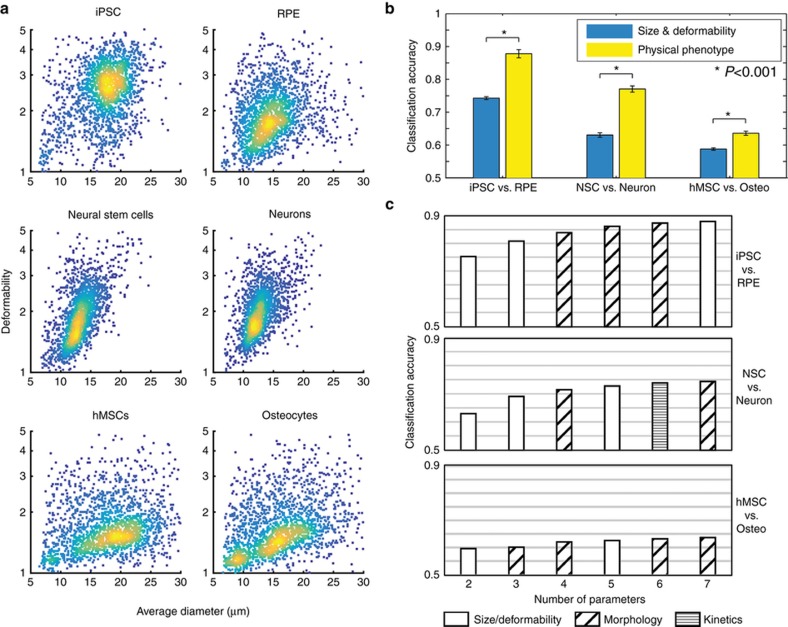
Cell classification improves with morphology and kinetics parameters. (**a**) Scatter plots of cell size and deformability for induced pluripotent stem cells (iPSCs), retinal pigmented epithelial cells (RPEs), neural stem cells (NSCs), neurons, mesenchymal stem cells (hMSCs), and osteocytes. Each pairing (iPSC/RPE, NSC/neuron, hMSC/osteocyte) represents a differentiated cell type and its progenitor stem cell. In the cases of NSCs vs. neurons and hMSCs vs. osteocytes, there are not clear changes in cell size and deformability, suggesting that classification of these cell types based on these parameters alone would be difficult. *N*=5000 for each scatter plot. (**b**) Classification accuracies of support vector machines (SVMs) trained on each of the three cell type pairs. In each case, SVMs were supplied with either the full physical phenotype or just size and deformability. In all cases, the addition of parameters improved classification accuracy. (**c**) SVMs were trained starting with size and deformability followed with the sequential addition of five additional parameters from the four categories (size, deformability, morphology, and kinetics parameters). The parameters are listed from left to right in order of importance in improving classification accuracy in a cumulative manner. In all cases, SVMs were trained using 5000 randomly sampled cells of each cell type.

We first investigated the repeatability and robustness of the new metrics extracted from the high-speed videos of cell deformation. We found that similar to the previously introduced deformability and size (Figure 2a), metrics of morphology and deformation kinetics possessed similar characteristics run to run, independent of slight changes in lighting and flow conditions. For NSCs, the median max deformability had a mean of 1.85 and a coefficient of variance (cv) of 0.15 across nine replicates (three technical replicates from three separate biological replicates). The average median cell size was 13.1 μm with a cv of 0.69. The surface roughness morphology parameter had an average median value of 141.4 and a cv of 0.14. The average relaxation rate, a deformation kinetics parameter, was the least reproducible with a mean value of −0.09 with a cv of 0.21 (Supplementary Table S2).

To investigate the utility in identifying a cell state with these additional parameters beyond deformability and size, SVMs were trained with the full physical phenotype or with only cell size and maximum deformation. In each case, SVMs that were trained with the full physical phenotype outperformed those trained with only size and deformation (Figure 2b). The improvements to classification accuracy were not uniform and depended on the comparison populations. The comparison that benefited the most from the addition of new physical phenotype parameters was NSCs vs. neurons, with a 14-percentage point improvement, followed by iPSCs vs. RPEs, with a 13.5-percentage point improvement. The improvement for the comparison of hMSCs vs. osteocytes was the smallest at 4.8 percentage points. In each of the comparisons, the data represented pooled results from three or more biological replicates each with three or more technical replicates.

**Figure 3:**
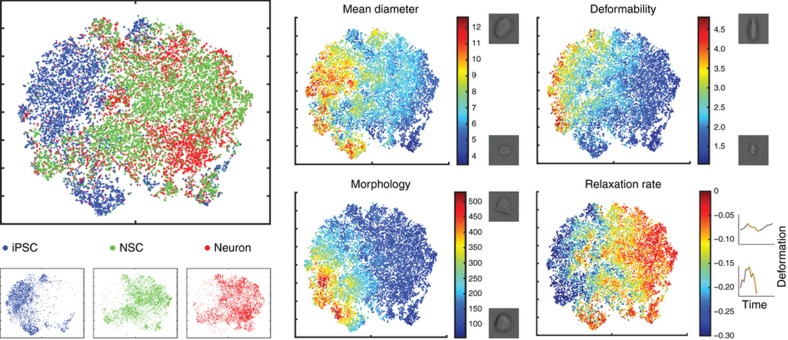
Visualization of physical phenotypic spaces occupied by iPSCs, NSCs, and neurons based on their 21 parameters. (**a**) viSNE is used to produce a 2D projection of the physical phenotypic space. These cells represent different points on the spectrum of differentiation. Separation in this projection indicates differences in physical phenotype. (**b**) The 2D projection from **a** recolorized according to a parameter from cell size, deformability, deformation kinetics or morphology. The resulting scatter plots demonstrate how these physical properties differ within cell populations and how they change during the differentiation process. 2D, two dimensional; iPSCs, induced pluripotent stem cells; NSCs, neural stem cells; viSNE, visual interactive stochastic neighbor embedding.

As shown above, m-DC generates high-dimensional information from individual cells; despite its benefits, data interpretation, and decision making from multi-dimensional data can be challenging. As such, we adopted viSNE to visualize the physical phenotypes of iPSCs, NSCs, and neurons without deteriorating the power of single-cell analysis (Figure 3). viSNE utilizes a *t*-distributed stochastic neighbor embedding algorithm to generate a scatter plot using all pairwise distances in a high-dimensional data set; as such, viSNE provides a biaxial scatter plot that best preserves the projection of the multidimensional physical phenotypic space at a single-cell level. The resulting projection shows that the three cell types occupy generally separable spaces, with some overlap. iPSCs showed the smallest degree of overlap with the other cell populations. NSCs and neurons exhibited a higher degree of overlap, though there were still spaces uniquely occupied by cells in either population.

By relabeling the viSNE projection using a parameter from each of the four broad categories (size, deformability, morphology, and kinetics), a connection to physical changes between the cells during differentiation can be better discerned. This reveals general characteristics of the cell populations and changes that occur during differentiation. In general, iPSCs are larger and more deformable than the more differentiated NSCs and neurons. In addition, iPSCs have a higher average relaxation rate and higher surface roughness than the other cell populations (Figure 3). Comparing NSCs and neurons, we can see differences as well. NSCs are larger and less deformable than neurons with higher surface roughness and lower average rates of relaxation.

## DISCUSSION

m-DC improves upon the previously published deformability cytometry system by expanding the parameters extracted by our analysis algorithm to include morphology and deformation kinetics metrics in addition to new cell size and deformability metrics. The new parameter set, the physical phenotype of a cell, consists of 19 new parameters for a total of 21. We demonstrated that the new metrics contain useful information by measuring the physical phenotypes of several pluripotent cells and their differentiated descendants. We then used the physical phenotypes to train SVMs that revealed that the new parameters improve the classification of these cell types in comparison to SVMs trained only on average cell diameter and maximum deformation. The improvement in classification accuracy indicates that the new parameters capture biologically relevant information that can aid in the identification of these cell populations. It is worth noting that although the classification accuracy increased across all of the cell comparisons, the improvements were not uniform and were cell-type dependent.

The highest observed cell classification accuracy was 87.4% with iPSCs and RPEs. Although the accuracy is not yet high enough for confident determination of all cells in a mixed cell population, our results indicate the potential of the m-DC tool for identifying pluripotent cells within a population of more differentiated cells such as neural stem cells or retinal pigment epithelial cells, and the approach may be more generally applicable to the characterization of the level of remaining pluripotent cells remaining in other cultures, especially given the unique biophysical features for these very phenotypically plastic cells.

We also performed sequential training of SVMs to determine which five parameters were most useful in improving classification accuracy in each comparison. Not surprisingly, the five parameters that emerged as most important in each comparison were dependent on cell type.

We discovered that morphological parameters and additional deformability metrics were generally important in improving accuracy; however, kinetics parameters were less so, only improving classification between NSCs and neurons. We ranked the physical phenotype parameters by incrementally increasing the number of parameters supplied to an SVM and instructing the SVM to select the parameter that best improved classification at each iteration. This analysis revealed that morphologic parameters as well as new size and deformability measurements were important in improving classification accuracy for most cell populations (Figure 2c and Supplementary Table S3). New size parameters compute the cell area instead of the cell diameter, which can be affected by differences in cell morphology such as shape. Thus, the new size parameters may be able to more accurately capture the differences in cell size that can be seen in Figure 2. The new deformability parameters also corrected for cell morphology by measuring the increase in the aspect ratio during the deformation process relative to the aspect ratio prior to deformation instead of using a circle as the baseline reference. In addition, a kinetics parameter, the mean relaxation rate, was useful in improving the classification of NSCs and neurons but was not one of the top five metrics that added additional information beyond deformability and size for classification for the other cell types tested.

Case-specifically, in the comparison between iPSCs and RPEs, the important parameters were morphology and size metrics. The morphology parameters encompass both surface roughness and cell shape, and the size metrics evaluate the area of a cell instead of the previously used size parameter (average diameter). Morphology parameters compare the actual distance from the cell membrane to the cell centroid as a function of radial angle with the moving average of the distance. The surface roughness parameters use a short moving average (5°), and the cell shape parameters use a longer moving average (30°). The importance of the surface roughness and cell shape parameters indicates that there are important differences in both of these types of morphologies between the two cell populations, perhaps due to cortical actin differences, which lead to more ruffling or blebbing. In addition, it is likely that the area measurements capture the size of iPSCs better when the irregular shape of the cells interferes with diameter measurements.

In the comparison between NSCs and neurons, the important parameters also included cell area, surface roughness, and cell shape. Notably, the important parameters also included a deformation kinetics parameter, the average relaxation rate. The kinetics parameters are computed based on the cell aspect ratio as a function of time during the deformation process. Increases in the aspect ratio are recorded as deformations, while decreases are recorded as relaxations. Thus, the importance of the average relaxation rate metric indicates that the time-dependent characteristics of the deformation process contain important information about cell phenotypes. It should be noted that this time-dependent metric was found to have a larger inter-trial measurement variation as discussed above with a cv up to 2–3-fold higher than that for deformability measures, which may be one reason for the reduced importance compared to that of the other metrics in the classification of cells.

Lastly, the comparison between hMSCs and osteocytes benefited as well from cell area, surface roughness, and cell shape. Interestingly, a morphology parameter that examines the aspect ratio of the cell prior to deformation was also important. This serves as another measurement of cell shape and indicates that morphology is an important distinguishing factor for these cell types.

The analysis of each of these comparisons indicates that some physical phenotype parameters such as cell area and morphology are very important factors in cell classification. However, the differences in each comparison, such as the use of a kinetics parameter in the NSC vs. neuron comparison, reveals that there is no essential set of physical parameters that defines cell types and that many of the different physical phenotype parameters have the potential for being important factors in cell classification.

A hallmark of neuron differentiation is the development of a polarized cell structure with axons and dendrites. Studies have shown that the neural differentiation process involves significant reorganization of the cytoskeleton including actin, intermediate filaments, and microtubules^25,26^. A key biomarker for neural stem cells is nestin, an intermediate filament protein whose expression is tightly regulated during differentiation^27^. Furthermore, microtubule organization is particularly important both for maintaining the polarized structure of neurons as well as facilitating their activity^28,29^. These changes to cytoskeletal structure may contribute to the observed decrease in deformability of NSCs and neurons relative to iPSCs as well as the observed importance of cell morphology when classifying NSCs and neurons (Figure 2).

Previous work on hMSCs and osteogenesis has demonstrated that cytoskeletal changes occur during the differentiation process. Pronounced cell shape changes occur, mediated in part by extensive reorganization of actin into thick bundles at the cell periphery^30,31^. In addition, previous work has also demonstrated that MSCs undergo changes in mechanical properties such as Young’s modulus during osteogenesis, although the nature of the change can be method dependent^32,33^. These changes in cell shape due to actin reorganization may help explain the differences in cell morphology observed in m-DC (Figure 2). The ambiguous results of previous mechanical measurements comparing hMSCs and osteocytes may also help explain the relative unimportance of deformability when distinguishing the two cell types.

There have been studies demonstrating changes in expression of several genes and transcription factors (e.g., cathepsin D, Pax 6, calbindin, PKC-a, and Mitf) during pluripotent stem cell differentiation^34,35^; many of these contribute to the organization of the cytoskeleton through microfilaments, intermediate filaments, and/or microtubules^36–43^. For example, upregulation of Pax6 or PKC-a during differentiation may stabilize the cytoskeleton^44,45^. Furthermore, loosely organized heterochromatin and/or abundant euchromatin modifications have been observed in pluripotent cells compared to that observed in differentiated cells^46–51^. These changes to cytoskeletal and nuclear structure may contribute to the decrease in deformability and overall change in physical phenotype observed in RPEs compared to that in iPSCs (Figure 2).

Further improvements to the measurement of physical phenotype can be made in the future by focusing on decoupling the effects of cell size on deformability and deformation rate parameters. In the previously described SVM experiments, size information was provided to the SVMs so that deformability and kinetics could be considered in the context of cell size. However, direct comparisons of cell deformability and kinetics will benefit from correcting for cell size.

Finally, the visualization of the physical phenotypes of iPSCs, NSCs, and neurons revealed that the three cell types occupy generally separable spaces, confirming that changes in physical phenotype occur throughout the differentiation process. Further exploration revealed changes in all four broad categories of physical parameters. Thus, we have used m-DC to begin the process of mapping the physical changes that occur during differentiation. A general trend found in moving along the spectrum from less to more differentiated was increasing stiffness and more circular cell shapes in suspension. This may be linked to higher cortical tension, and these maps can be useful in understanding the process of differentiation and the importance of physical properties. They can also aid in the detection of subpopulations and provide context to physical changes that occur in other biological processes such as neoplasia.

## CONCLUSIONS

In this report, we demonstrated m-DC, an improvement to the previously described deformability cytometry platform by adding two new categories of physical parameters: cell morphology and deformation kinetics. In conjunction with size and deformability metrics, the new parameters produce a description of a cell’s physical phenotype. Using SVMs, we demonstrated that the physical phenotype improves classification of pluripotent stem cells and their differentiated descendants. In addition, we showed that the new categories of parameters are important contributors to the improved classification accuracy. Finally, we demonstrated how the physical phenotype can be visualized and used to explore the physical changes that occur during the differentiation process. m-DC is a high-throughput and label-free method for analyzing the physical properties of cells. This technique opens the door to label-free assays of differentiation progression with applications in stem cell therapy. Furthermore, the biophysical maps produced by measurements of stem cells and their descendants provide a tool for studying the role of differentiation in other biological processes such as cancer. Ultimately, such approaches can deepen our understanding of subtle changes to cell phenotypes and their implications in physiological processes.
