# Supplementary material for: High-throughput physical phenotyping of cell differentiation
Source: Microsyst Nanoeng. 2017 May 8;3:17013. doi: 10.1038/micronano.2017.13 (PMC6445007; doi:10.1038/micronano.2017.13)
Supplement: Supplementary Information [file micronano201713-s1.pdf]

Supplementary file

High-throughput physical phenotyping of cell differentiation

Jonathan Lin[1,*], Donghyuk Kim[1,*], Henry Tse[2], Peter Tseng[3], Lillian Peng[1], Manjima Dhar[1], Saravanan Karumbayaram[4] and Dino Di Carlo[1,5,6,7]

Microsystems & Nanoengineering (2017) **3,** 17013; doi:10.1038/micronano.2017.13; Published online: 8 May 2017

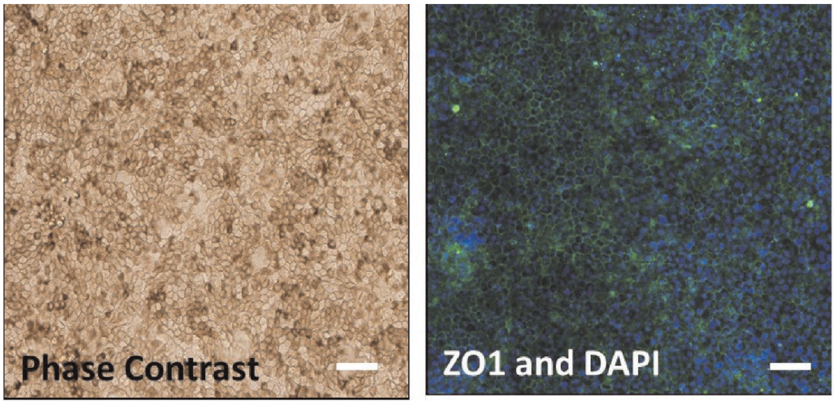

Figure S1 Retinal pigmented epithelial (RPE) cell characterization. Phase contrast images showed the normal pigmented structure of an RPE monolayer. RPEs were immunostained for tight junction protein, ZO1 (using rabbit anti-ZO1, Invitrogen, shown in green) which is a common marker for RPEs, and their nuclei (4′,6-diamidino-2-phenylindole or DAPI shown in blue). The procedure is as follows: a monolayer of RPE cells were fixed in 4% paraformaldehyde (PFA) for 15 min and permeabilized with 0.1% triton X-100 for 10 min. Cells were blocked in 10% goat serum. Scale bar: 100 μm (Ref. 1).

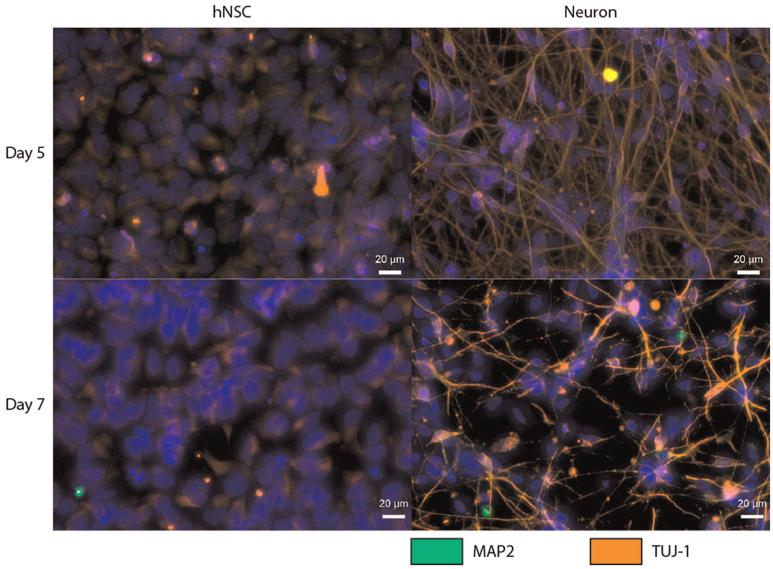

Figure S2 Human neural stem cells (hNSCs) and neurons stained using immunohistochemistry for neural markers. hNSCs and neurons were fixed in 0.2% paraformaldehyde for 10 min, permeabilized in 0.04% Triton X-100 for 10 min, and immuno-stained with anti-MAP2 (EMD Millipore MAB5326A4) and anti-TUJ-1 (Abcam ab18207), two neural cell markers. 5% goat serum was used as a blocking agent. Neurons at 5 and 7 days post-plating show high levels of TUJ-1 expression and demonstrate clear neural morphology with extensive neural processes. Nuclear staining was performed with DAPI (Thermo Fisher).

[1]Department of Bioengineering, University of California, Los Angeles, CA 90095, USA; [2]CytoVale Inc., 384 Oyster Point Blvd. #7, South San Francisco, CA 94080, USA; [3]Department of Biomedical Engineering, Tufts University, Medford, MA 02155, USA; [4]Eli & Edythe Broad Center of Regenerative Medicine & Stem Cell Research, Los Angeles, CA 90095, USA; [5]California NanoSystems Institute, Los Angeles, CA 90095, USA; [6]Jonsson Comprehensive Cancer Center, Los Angeles, CA 90095, USA and [7]Department of Mechanical Engineering, University of California, Los Angeles, CA 90095, USA
Correspondence: Dino Di Carlo (dicarlo@seas.ucla.edu)
*These authors contributed equally to this work

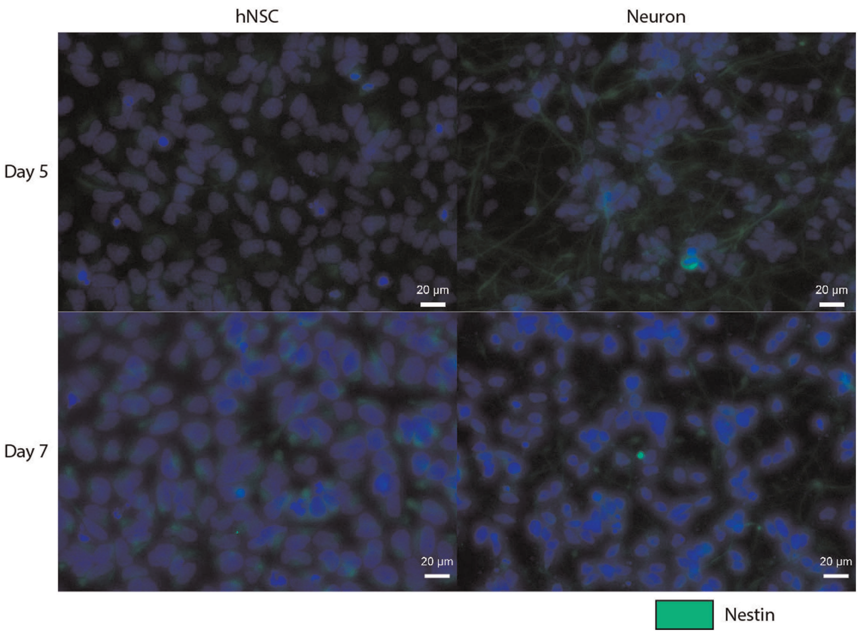

Figure S3 hNSCs and neurons staining for nestin. hNSCs and neurons were fixed, permeabilized, and immuno-stained with anti-nestin (EMD Millipore clone 10C2 MAB5326A4) a neural stem cell marker. hNSCs at 5 and 7 days following seeding show nestin expression. Neurons at day 5 show nestin expression with clear neural morphology and neurons at day 7 show decreasing levels of nestin expression. Nuclear staining was performed with DAPI (Thermo Fisher).

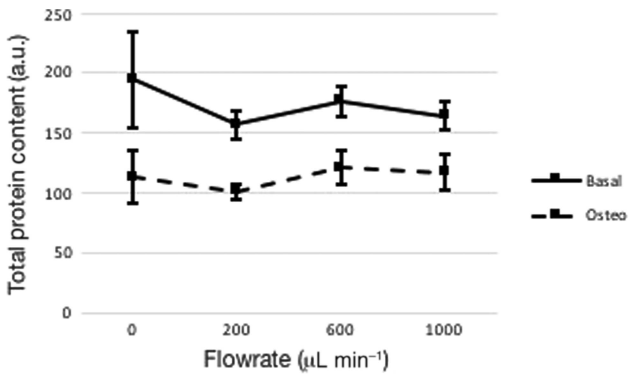

Figure S4 hMSC and osteocyte viability 1 week following DC measurements. hMSCs were run through the DC device at varying flowrates to determine the effect of DC on cell viability. Samples with flowrates of 0 μL min^{-1} were not exposed to stretching or any flow. Following treatment, the cells were seeded at equal density and kept in either MesenPRO RS medium or osteogenic medium for 1 week. After 1 week, a BCA assay (Pierce) was performed to determine total protein concentration which was used as a proxy for cell viability and proliferation. There is no clear effect of DC treatment on hMSC viability. Each data represents 4 replicates and error bars represent a single standard deviation.

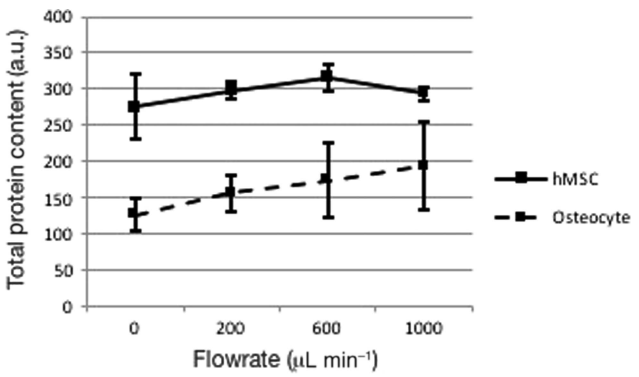

Figure S5 hMSC and osteocyte viability 2 weeks following DC measurement. hMSCs were treated as described in Figure S4. After 2 weeks, a BCA assay (Pierce) was performed to determine total protein concentration which was used as a proxy for cell viability and proliferation. There is no clear effect of DC treatment on hMSC viability. Each data represents 4 replicates and error bars represent a single standard deviation.

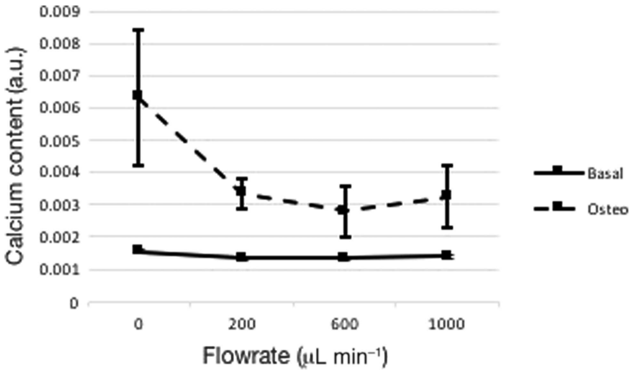

Figure S6 hMSC and osteocyte calcium content 2 weeks following DC measurement. Calcium content, an osteogenesis marker, was assayed (QuantiChrom, BioAssay Systems) and normalized against total protein content at 2 weeks for the hMSCs and osteocytes described in Figure 6. There is no clear effect of DC treatment on the differentiation of hMSCs. Error bars represent 1 standard deviation.

REFERENCE

1 Maminishkis A, Chen S, Jalickee S *et al.* Confluent monolayers of cultured human fetal retinal pigment epithelium exhibit morphology and physiology of native tissue investigative. *Investigative Ophthalmology & Visual Science* 2006; **47**: 3612–3624.

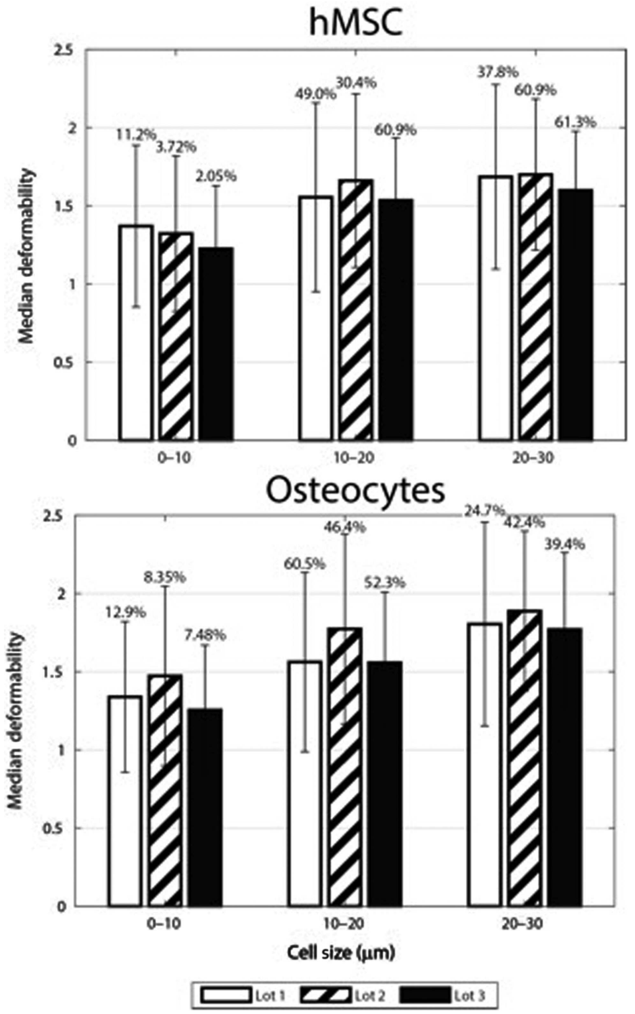

Figure S7 Lot variation in hMSCs. Three batches of adipose-derived stem cells were purchased from Thermo Fisher and measured with several replicates using DC. Here, the median deformability is plotted as a function of cell size for both hMSCs and the osteocytes that were differentiated from them. Error bars represent a standard deviation and numbers above bars represent the percentage of cells that fall within the size range. Although cells within a given size range have similar deformability across multiple batches of hMSCs, the percentage of cells that fell within the size bins varied between batches. Over 20 000 events were collected for each batch of hMSCs. This demonstrates the ability to detect batch-to-batch variation in stem cell properties that is enabled by high-throughput, single-cell measurements.

Table S1 Definition of 21 parameters

Parameter	Description
Average Size	Average cell diameter. The average cell diameter is computed for each frame prior to the beginning of the deformation process. For each frame, the cell boundary is detected and the diameter is measured in the vertical direction by taking the average of 61 diameters between the angles 60 to 120 degrees, each separated by 1 degree of rotation. These average diameters are then averaged again for an overall pre-deformation cell diameter.
Area	Crude approximation of average cell area. The cell boundary is detected for each frame prior to the beginning of the deformation process. The distance from the cell boundary to the cell centroid is computed as a function of angle with 360 data points each separated by 1 degree of rotation. The sum of these distances is computed and then averaged over all pre-deformation frames.
Area 2	Average cell area. The cell boundary is detected for each frame prior to the beginning of the deformation process. The distance from the cell boundary to the cell centroid is computed as a function of angle with 360 data points each separated by 1 degree of rotation. The distance data is then smoothed using first order Savitzky-Golay filter with a window size of 17. The resulting data is converted back to Cartesian coordinates and the area of the cell is determined using MATLAB's polyarea function.
Final Aspect Ratio	Peak aspect ratio of the cell during the deformation process. Aspect ratio is defined as the long axis of the cell divided by the short axis of the cell. The two axes are defined such that one is vertical (between 70 and 110 degrees) and one is horizontal (between − 20 and 20 degrees).
Compensated Aspect Ratio	Aspect ratio measurement that attempts to control for non-unity cell aspect ratio prior to deformation. Compensated aspect ratio is the difference between the final aspect ratio and the initial aspect ratio.
Morphology 1A	The cell boundary is detected for each frame prior to the beginning of the deformation process. The distance from the cell boundary to the cell centroid is computed as a function of angle with 360 data points each separated by 1 degree of rotation. This yields cell radius as a function of angle which is used to compute a moving average of length 5 degrees. The moving average is subtracted from the raw radius data and the absolute value of the result is integrated over the entire cell (360 degrees).
Morphology 1C	The cell boundary is detected for each frame prior to the beginning of the deformation process. The distance from the cell boundary to the cell centroid is computed as a function of angle with 360 data points each separated by 1 degree of rotation. This yields cell radius as a function of angle which is used to compute a moving average of length 5 degrees. The moving average is compared to the raw cell data and the number of cross over events between the two functions is counted.
Morphology 2A	Same as Morphology 1A but over 15 degrees for the moving average.
Morphology 2C	Same as Morphology 1C but over 15 degrees for the moving average.
Morphology 3A	Same as Morphology 1A but over 30 degrees for the moving average.
Morphology 3C	Same as Morphology 1C but over 30 degrees for the moving average.
Initial Aspect ratio	Peak aspect ratio of the cell prior to the deformation process. Aspect ratio is defined as the long axis of the cell divided by the short axis of the cell. The two axes are defined such that one is vertical (between 70 and 110 degrees) and one is horizontal (between − 20 and 20 degrees).
Cell size and morphology	Combined measurement of surface roughness and cell size. Prior to deformation, cell diameter is measured in the vertical direction (between 60 to 120 degrees). Additionally, the distance from the cell boundary to the cell centroid is computed as a function of angle with 360 data points each separated by 1 degree of rotation. The cell diameter is then added to the sum of the absolute value of the derivative of the cell boundary distance.
Max Deformation Rate	Cell aspect ratio is recorded as a function of time during the deformation process. This function is differentiated to produce the rate of aspect ratio change as a function of time. This parameter represents the maximal positive rate of aspect ratio change.
Initial Deformation Rate	Cell aspect ratio is recorded as a function of time during the deformation process. This function is differentiated to produce the rate of aspect ratio change as a function of time. This parameter represents the first recorded positive rate of aspect ratio change.
Average Deformation Rate	Cell aspect ratio is recorded as a function of time during the deformation process. This function is differentiated to produce the rate of aspect ratio change as a function of time. This parameter represents the average of all recorded positive rate of aspect ratio change.
Max Relaxation Rate	Cell aspect ratio is recorded as a function of time during the deformation process. This function is differentiated to produce the rate of aspect ratio change as a function of time. This parameter represents the maximal negative rate of aspect ratio change.
Initial Relaxation Rate	Cell aspect ratio is recorded as a function of time during the deformation process. This function is differentiated to produce the rate of aspect ratio change as a function of time. This parameter represents the first recorded negative rate of aspect ratio change.
Average Relaxation Rate	Cell aspect ratio is recorded as a function of time during the deformation process. This function is differentiated to produce the rate of aspect ratio change as a function of time. This parameter represents the average of all recorded negative rate of aspect ratio change.

Table S1. (Continued)

Parameter	Description
Deformation Rate before maximum deformation	Cell aspect ratio is recorded as a function of time during the deformation process. This function is differentiated to produce the rate of aspect ratio change as a function of time. This parameter represents the positive rate of aspect ratio change that occurs just prior to the maximum measured aspect ratio.
Wobble	Cell aspect ratio is recorded as a function of time during the deformation process. This function is differentiated to produce the rate of aspect ratio change as a function of time. A second order polynomial is then fit to this rate of change function and the root mean square of the residuals is reported.

Table S2 Systemic technical and biological variability in biophysical measurements of neural stem cells

Biological replicate	Deformability CV	Cell size CV	Surface roughness CV	Relaxation rate CV
1	0.015	0.009	0.018	0.013
2	0.032	0.010	0.021	0.070
3	0.013	0.003	0.037	0.039
All	0.167	0.079	0.158	0.237

This table compares the variability due to the m-DC system with the biological variability of neural stem cells. The first three rows of the table contain the coefficients of variance for the average median values of 4 physical phenotype parameters for each of 3 biological replicates. Each statistic represents the results of 3 technical replicates and gives an estimate of the variability of the m-DC measurement itself. The final row of the table contains the coefficients of variance for the average median values of the same parameters for the overall dataset. Each statistic describes the results of 3 biological replicates and gives an estimate of the biological variability of neural stem cells.

Table S3 Repeatability of relative importance of physical phenotype parameters

iPSC vs. RPE	3	4	5	6	7
Replicate 1	Area	Cell size and morphology	Morphology: M1A	Morphology: M3A	Area 2
Replicate 2	Area	Morphology: M1C	Morphology: M3C	Area 2	Compensated aspect ratio
Replicate 3	Area	Morphology: M1C	Cell size and morphology	Morphology: M1A	Morphology: M3A
NSC vs. Neuron					
Replicate 1	Area 2	Morphology: M3A	Area	Average relaxation rate	Cell size and morphology
Replicate 2	Area 2	Morphology: M3A	Average relaxation rate	Morphology: M1C	Area
Replicate 3	Area 2	Morphology: M3A	Average relaxation rate	Area	Morphology: M2A
hMSC vs. Osteocyte					
Replicate 1	Cell size and morphology	Morphology: M2A	Area 2	Compensated aspect ratio	Morphology: M1A
Replicate 2	Morphology: M2A	Cell size and morphology	Morphology: M1A	Morphology: M2C	Average deformation rate
Replicate 3	Morphology: M1C	Cell size and morphology	Area	Morphology: M3C	Initial aspect ratio

This table depicts three replicates of the SVM-based experiments to determine the relative importance of physical phenotype parameters. In these experiments, SVMs were initially supplied with average cell size and deformability. From there, parameters were iteratively added to the SVM, maximizing the improvement of classification accuracy at each iteration (see Materials and Methods). Replicates of these experiments demonstrate that some parameters are robustly important for distinguishing certain pairings of cells (e.g., cell area for iPSC vs. RPE) while other parameters are seemingly interchangeable (e.g., Morphology M3A and M3C in iPSC vs. RPE). This confirms that parameters can often be correlated and that there is not a clear minimum set of parameters capable of describing all cell types.
